# Alkalized lidocaine in endotracheal tube cuff inflation in patients undergoing thyroidectomy surgery: a clinical trial

**DOI:** 10.1590/1806-9282.20240740

**Published:** 2024-11-11

**Authors:** Gaudencio Barbosa, Geraldo José Coelho Granja, Caio Marcio Barros de Oliveira, Plínio da Cunha Leal, Marcelo Souza de Andrade, Ed Carlos Rey Moura

**Affiliations:** 1 Universidade Federal do Maranhão, Postgraduate Program in Adult Health – São Luís (MA), Brazil.; 2 Universidade Federal do Maranhão, Department of Medicine – São Luís (MA), Brazil.

**Keywords:** Thyroidectomy, Endotracheal intubation, Lidocaine

## Abstract

**OBJECTIVE:**

The aim of the study was to compare the postoperative effects of endotracheal tube cuff inflation with alkalized lidocaine in patients undergoing thyroidectomy surgery.

**METHODS:**

This is a randomized, double-blind clinical trial between August 2020 and August 2022 at the Hospital São Domingos, São Luís, Maranhão, Brazil. Patients over 18 years who underwent thyroidectomy of both sexes, American Society of Anesthesiologists (ASA) I or ASA II, were included. Patients with difficult orotracheal intubation, smoking, cuff rupture during orotracheal intubation, heart, lung, or neuropathies, previous surgery of the larynx or trachea, with risk of aspiration of gastric contents, or with the need to use a nasogastric tube were excluded. Patients were randomly selected and divided into the control group, whose cuff was filled with 0.9% saline solution, and the alkalinized lidocaine group, where the cuff was filled with 2% lidocaine and 8.4% sodium bicarbonate.

**RESULTS:**

The control group had higher systolic blood pressure [137 (94–183) mmHg] medians after extubation than the alkalinized lidocaine group [127 (82–189) mmHg] (p=0.03). The same was observed in heart rate values [control group: 86 (62–120); alkalinized lidocaine group: 80 (53–120)] (p=0.034). The alkalinized lidocaine group showed a significant increase in the ability to sustain phonation in the 24 h postoperatively, from 82.0 to 98.0% (p=0.031).

**CONCLUSION:**

There was no protective effect of the use of alkalinized lidocaine on the sensation of swallowing and complaints after thyroidectomy surgery. There was a significant improvement in hemodynamic response in the intervention group after extubation.

## INTRODUCTION

To perform surgical procedures due to suspected or confirmed malignant dysfunctions and benign, in the presence of obstructive symptoms, aesthetic problems, and hyperthyroidism^1^, patients are submitted to general anesthesia and receive orotracheal intubation (OTI), which is a medical procedure, invasive and of advanced support that allows the control of the airways, preserving the patient's breathing^2^.

Events resulting from extubation are frequent and usually related to the parasympathetic system, such as coughing, which can result in wound dehiscence^3^. Extubation is performed before the end of anesthesia to reduce events and discomfort, combined with intravenous opioids or lidocaine, lidocaine spray, gel, or ointment applied to the trachea^4,5^.

Studies indicate that lidocaine inserted into the endotracheal tube (ETT) cuff inflation decreases pain, laryngeal reflex, and cough^5,6^. Its mechanism of action involves drug diffusion through the cuff wall, blocking local receptors and thus reducing discomfort caused by cuff contact with the tracheal mucosa, with increased effectiveness when associated with sodium bicarbonate (8.4%) or heated.

Therefore, the study aimed to compare the postoperative effects of endotracheal tube cuff inflation with alkalized lidocaine (AL) in patients undergoing thyroidectomy surgery.

## METHODS

### Trial design

This study is a randomized, double-blind clinical trial from August 2020 to August 2022 at the Hospital São Domingos, São Luís, Maranhão, followed by the Consolidated Standards of Reporting Trials (CONSORT).

### Ethical information

The study followed the Declaration of Helsinki, was approved by the Ethics Committee of the Federal University of Maranhão (CAAE: 20790619.8.0000.5085), and was registered in the Brazilian Registry of Clinical Trials (RBR-4rvdvfk) and Universal Test Number U1111-1284-8804. Informed consent was obtained from all individual participants included in the study.

### Participants

Patients over 18 years who underwent thyroidectomy of both sexes, American Society of Anesthesiologists (ASA) I or ASA II, were included. Patients with difficult OTI, smoking, cuff rupture during OTI, heart, lung, or neuropathies, previous surgery of the larynx or trachea, with risk of aspiration of gastric contents, or with the need to use a nasogastric tube were excluded.

### Interventions

Laryngoscopy was performed with a number 3 or 4 Macintosh blade, and the trachea was intubated with an orotracheal tube number 7.0 or 7.5 (women) and 7.5 or 8.0 (men). The anesthesia used in the study was of the general type (total venous) with induction made by propofol 2 mg/kg, fentanyl 3–5 mg/kg, and rocuronium 0.6 mg/kg. Propofol+remifentanil, aiming at bispectral index 40–60, were used for maintenance. Dipyrone 2g+ondansetron 8 mg+sugammadex 4 mg/kg were administered before extubation. The procedure was performed by resident anesthetists and their supervisors.

Cuff inflation followed the following criteria:

Control group (CG): 6 mL of 0.9% saline solution.

Alkalinized lidocaine group (ALG): 2 mL of 2% lidocaine without vasoconstrictor and 4 mL of 8.4% sodium bicarbonate.

### Outcomes

The primary outcome is the result of the assessment of physiological reflexes to extubation, such as cough, time to awakening, speech recovery, and perioperative blood pressure levels.

The secondary outcome refers to swallowing sensation, ability to sustain phonation and to sustain adequate volume, as well as complaints of dysphonia, emesis, vocal fatigue, hemoptysis, airway inflammation, fullness, hoarseness, foreign body sensation, whispering, coughing, fullness, hoarseness, and foreign body sensation.

### Assessment and data collection instruments

Data were collected from medical records and anesthetic evaluation forms and recorded on a form with closed questions, and hemodynamic patterns, swallowing sensation, and postoperative complaints were also evaluated.

### Randomization

The patients were randomly assigned numbers from 1 to 100, with 50% of the numbers belonging to the CG, in which the cuff was filled with 0.9% saline, and 50% belonging to the intervention group (ALG), in which the cuff was filled with 2% lidocaine and 8.4% sodium bicarbonate. The numbers assigned to each group were drawn using the Research Randomizer application. This study was blinded to the patient and surgeon. The anesthetist was responsible for whether or not to apply AL to the cuff.

### Sample size

Considering a proportion confidence interval of 35% of the occurrence of cough and odynophagia, with an average of 12 procedures per month, a confidence interval of 95%, an alpha of 5%, a power equal to 80%, and considering a 10% chance of loss, it was estimated that 100 patients were evaluated.

### Statistical methods

Data were analyzed using SPSS 21.0^®^. Normality was verified using the Shapiro-Wilk test. Differences in means or median were compared with the Student's t-test or Mann-Whitney test. For categorical variables, the chi-square test was applied. Differences were considered significant at p<0.05.

## RESULTS

**Figure 1:**
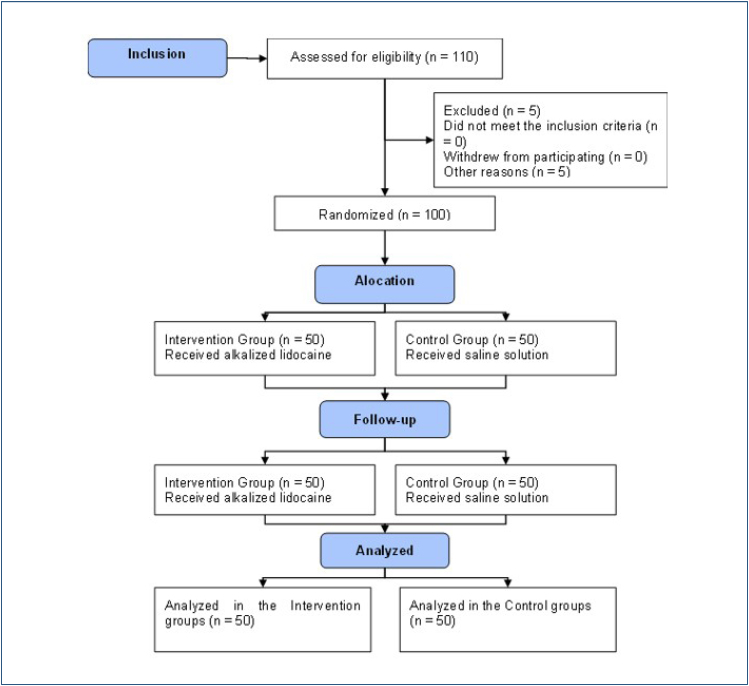
The CONSORT flowchart.

A total of 100 patients were included in the study, 50 in the CG and 50 in the ALG, as seen in the CONSORT flowchart (Figure 1).

Women were predominantly observed (78.0 and 88.0%, CG and ALG, respectively), with 41 and 50 years of age (CG: 28.0 and ALG: 34.0%), with mean ages of 48.8±15.1 and 49.9±14.0 years, in that order, both groups had a high proportion of elderly (24.0 and 28.0%, CG and ALG). Additionally, 52.0 and 58.0% of residents were from the state capital, brown (60.0 in the CG and 42.0% in the ALG), and married (60.0 in the CG and 58.0% in the ALG). More individuals observed had completed high school in the CG (34.0%), while in the ALG, more individuals observed had completed higher education (34.0%). No statistically significant differences were found between the groups (Table 1).

**Table 1:** Sociodemographic characterization.

Variables		Group		p-value χ^2^
		CG	ALG	
		n (%)	n (%)	
Sex				
	Female	39 (78.0)	44 (88.0)	0.183
	Male	11 (22.0)	6 (12.0)	
Age (years)				
	21–30	4 (8.0)	4 (8.0)	0.860
	31–40	12 (24.0)	8 (16.0)	
	41–50	14 (28.0)	17 (34.0)	
	51–59	8 (16.0)	7 (14.0)	
	Over 60	12 (24.0)	14 (28.0)	
	Md±SD	48.8±15.1	49.9±14.0	0.702€
Residence				
	Capital	26 (52.0)	29 (58.0)	0.920
	Metropolitan region	8 (16.0)	8 (16.0)	
	Upstate	15 (30.0)	12 (24.0)	
	Other state	1 (2.0)	1 (2.0)	
Race				
	Indigenous	1 (2.0)	1 (2.0)	0.211
	White	16 (32.0)	19 (38.0)	
	Brown	30 (60.0)	21 (42.0)	
	Black	3 (6.0)	9 (18.0)	
Marital status				
	Single	15 (30.0)	15 (30.0)	0.988
	Married	30 (60.0)	29 (58.0)	
	Divorced	1 (2.0)	1 (2.0)	
	Widower	4 (8.0)	5 (10.0)	
Education				
	Illiterate	2 (4.0)	1 (2.0)	0.563
	Incomplete primary education	7 (14.0)	9 (18.0)	
	Complete primary education	7 (14.0)	3 (6.0)	
	Incomplete high school	1 (2.0)	1 (2.0)	
	Complete high school	17 (34.0)	11 (22.0)	
	Incomplete higher education	1 (2.0)	3 (6.0)	
	Complete higher education	10 (20.0)	17 (34.0)	
	Postgraduate	4 (8.0)	3 (6.0)	
	Master	1 (2.0)	2 (4.0)	

€ Student's t-test. CG: control group; ALG: alkalinized lidocaine group.

There were no statistically significant differences between the groups regarding anthropometric, clinical, and surgical characteristics. The CG and ALG groups presented approximate median body mass and BMI values, respectively, 68.4 and 67.8 kg and 26.2 and 26.6 kg/m². ASA assessment grade I was predominantly observed in the CG and ALG (64.0 and 62.0%) and Mallampati class II (40.0 and 36.0%, CG and ALG, respectively). Primarily, individuals performed total thyroidectomy, respectively, CG and ALG, 90.0 and 84.0%. The duration of anesthesia was slightly longer in CG (median=105 min) than in ALG (median=99 min), the surgical procedure lasted 71.5 min in CG and 74.5 min in ALG, and the recovery time was shorter in this group (median=1 min) than in CG (median=2 min). The speech recovery time shows the same median (median=2min). However, CG had a larger amplitude, with a maximum value of 18 min. No statistically significant differences in procedure times were found between the groups (Table 2).

**Table 2:** Anthropometric, clinical, and surgical characterization.

Variables		Group		p-value¥
		CG	ALG	
		Med (min–max)	Med (min–max)	
Body mass (kg)		68.35 (23.5–110)	67.5 (46.6–119)	0.767
Stature (m)		1.6 (1.4–1.8)	1.6 (1.4–1.8)	0.930
BMI (kg/m²)		26.15 (22.4–35)	26.6 (17.7–41.8)	0.937
ASA—n (%)				
	Grade I	32 (64.0)	31 (62.0)	0.836
	Grade II	18 (36.0)	19 (38.0)	
	Med (min–max)	1 (1–2.0)	1 (1–2.0)	0.837
Mallampati—n (%)				
	Class I	19 (38.0)	25 (50.0)	0.484
	Class II	20 (40.0)	18 (36.0)	
	Class III	10 (20.0)	7 (14.0)	
	Class IV	1 (2.0)	0 (0.0)	
	Med (min–max)	2 (1–4)	1.5 (1–3)	0.174
Thyroidectomy				
	Partial	5 (10.0)	8 (16.0)	0.372
	Total	45 (90.0)	42 (84.0)	
Duration of anesthesia (min)		105 (37–180)	99 (58–160)	0.746
Duration of surgery (min)		71.5 (49–123)	74.5 (32–116)	0.711
Time of wake up (min)		2 (0–67)	1 (0–30)	0.104
Time of regain speech (min)		2 (0–18)	2 (0–10)	0.919

CG: control group; ALG: alkalinized lidocaine group; BMI: body mass index; ASA: American Society of Anesthesiologists;

¥ Mann-Whitney.

The overall population had a median of 138 mmHg SBP (systolic blood pressure), 80 mmHg DBP (diastolic blood pressure), 101 mmHg MAP (mean arterial pressure), and an HR (heart rate) of 80.5 bpm. The evaluation 30 min after surgery also showed no differences between groups, with a median of 107 mmHg SBP, 64 mmHg and DBP, 80.5 mmHg, MAP, and HR of 69 bpm measured in the overall population. After extubation, the CG had a significantly higher median of SBP (124 mmHg) than the ALG (124 mmHg) (p=0.033), as well as HR (median CG=86 bpm; median ALG=80 bpm), with p=0.034. DBP and MAP had medians in the total population of 75.5 mmHg and 98 mmHg, respectively (p<0.05). Both groups had cough after extubation: 84.0 and 72.0% in CG and ALG, respectively (Table 3).

**Table 3:** Perioperative hemodynamic evaluation.

Hemodynamic evaluation		Group		p-value¥
		CG	ALG	
		Med (min–max)	Med (min–max)	
Before induction				
	SBP (mmHg)	139.5 (95–189)	137 (98–174)	0.482
	DBP (mmHg)	79 (63–105)	80 (60–108)	0.626
	MAP (mmHg)	101 (54–137)	99.5 (69–139)	0.710
	HR (bpm)	82 (58–103)	80 (57–105)	0.332
30 min after the start of surgery				
	SBP (mmHg)	106 (74–172)	107 (73–160)	0.930
	DBP (mmHg)	62.5 (30–128)	65.5 (39–109)	0.689
	MAP (mmHg)	79 (51–159)	83 (55–134)	0.760
	HR (bpm)	70 (45–95)	66.5 (51–100)	0.366
After extubation				
	SBP (mmHg)	137 (94–183)	127 (82–189)	0.033
	DBP (mmHg)	78 (52–141)	75 (51–102)	0.290
	MAP (mmHg)	99 (57–130)	95 (70–129)	0.444
	HR (bpm)	86 (62–120)	80 (53–120)	0.034
Cough—n (%)				
	Yes	42 (84.0)	36 (72.0)	0.148
	No	8 (16.0)	14 (28.0)	

CG: control group; ALG: alkalinized lidocaine group; SBP: systolic blood pressure; DBP: diastolic blood pressure; MAP: mean arterial pressure; HR: heart rate;

¥ Mann-Whitney.

The CG shows more subjects with the ability to maintain phonation in 2 h (88.0%) than the ALG (82.0%), which showed a significant increase (p=0.031) in 24 h postoperatively (94.0%) compared to 2 h. Individuals who were able to maintain adequate volume (72.0%) were observed more frequently in ALG than in CG (64.0), which again showed a significant increase in 24 h (86.0) (p=0.007). For dysphonia, vocal fatigue, hoarseness, and whispering, there was a significant decrease in both groups in 24 h compared to 2 h (p<0.05), but without significant differences between groups. In the ALG group, the percentage of subjects with foreign body sensation was higher (58.0%) than in the CG group (52.0%), which significantly decreased in 24 h (36.0%) (p=0.039). In both groups, there was a slight, non-significant increase in cough symptoms after 24 h compared to 2 h (Table 4).

**Table 4:** Swallowing sensation and complaints in the postoperative period of thyroidectomy with and without using alkalinized lidocaine in the inflation of the endotracheal tube cuff. São Luís, Maranhão, 2022.

Swallowing sensation and complaints		Group		p-value
		CG	ALG	
		n (%)	n (%)	
Ability to sustain phonation				
	2 h	44 (88.0)	41 (82.0)	0.401
	24 h	49 (98.0)	47 (94.0)	0.307
	p-value 2 vs. 24 h Φ	0.125	0.031	
Ability to sustain adequate volume				
	2 h	32 (64.0)	36 (72.0)	0.391
	24 h	43 (86.0)	41 (82.0)	0.585
	p-value 2 vs. 24 h Φ	0.007	0.302	
Dysphonia				
	2 h	20 (40.0)	16 (32.0)	0.405
	24 h	5 (10.0)	4 (8.0)	0.727
	p-value 2 vs. 24 h Φ	0.001	0.004	
Vocal fatigue				
	2 h	16 (32.0)	16 (32.0)	1,000
	24 h	3 (6.0)	3 (6.0)	1,000
	p-value 2 vs. 24 h Φ	<0.001	0.004	
Airway inflammation				
	2 h	22 (44.0)	22 (44.0)	1,000
	24 h	20 (40.0)	17 (34.0)	0.534
	p-value 2 vs. 24 h Φ	0.727	0.302	
Hoarseness				
	2 h	30 (60.0)	26 (52.0)	0.420
	24 h	8 (16.0)	9 (18.0)	0.790
	p-value 2 vs. 24 h Φ	<0.001	<0.001	
Foreign body sensation				
	2 h	26 (52.0)	29 (58.0)	0.546
	24 h	18 (36.0)	24 (48.0)	0.224
	p-value 2 vs. 24 h Φ	0.039	0.302	
Whisper				
	2 h	7 (14.0)	6 (12.0)	0.766
	24 h	0 (0.0)	0 (0.0)	
	p-value 2 vs. 24 h Φ	0.016	0.031	
Cough				
	2 h	8 (16.0)	5 (10.0)	0.372
	24 h	11 (22.0)	12 (24.0)	0.812
	p-value 2 vs. 24 h Φ	0.581	0.118	

Φ McNemar;

CG: control group; ALG: alkalinized lidocaine group.

## DISCUSSION

In this double-blind trial, there was no protective effect of using AL on swallowing sensation and complaints after thyroidectomy. However, lower hemodynamic values in the intervention group justify using local anesthesia to reduce the patient's hemodynamic parameters after extubation.

Predominated females, between 41 and 50 years of age, residents of the state capital, brown, married, with high school education being most observed in the CG and higher education completed in the ALG, ASA grade I in both the CG and ALG, Mallampati class II, and total thyroidectomies.

The predominance of women is reported in other studies^7,8^. Likewise, the range of age is compatible with other data found in the literature^8,9^. Also, a total thyroidectomy procedure^7^. The signs can be related to obstructive symptoms, aesthetic, hyperthyroidism, and suspicion of malignant neoplasm.

After extubation, the CG had higher medians for SBP than the ALG and HR. DAP and mean blood pressure showed similar medians between groups. Both presented cough after extubation, on average, for 24 h.

Similar to Navarro et al.^10^, comparing the pressures in tracheal tube cuffs filled with air or AL and evaluating the presence of cardiocirculatory and clinical manifestations, such as tolerance to the tracheal tube, cough, odynophagia, and hoarseness, and observed, after extubation, using 19.0 mL of 2% lidocaine to 1.0 mL of 8.4% sodium bicarbonate promoted a significant increase in SBP in the CG but not in DBP or HR.

Carrillo, López, and González^11^ also reported an increase in SBP using 2% at a dose of 1 mg/kg in the lidocaine group. The mean SBP was 115.9±15.15 mmHg before extubation and 123.02±15.91 mmHg after extubation, compared to the LG, 110+14.61 and 117.9±16.46 mmHg, respectively, before and after extubation, which demonstrates a significant increase in the CG (p=0.002).

Although no significant difference was observed between systolic or diastolic blood pressures in the moments before and shortly after tracheal extubation, in the study by Soares^12^ there was a lower increase in HR during extubation in the AL group, both at 1 and 0.5% lidocaine, when compared with whose cuffs were filled with air or saline solution.

Previous studies on the effects of AL on hemodynamics are highly variable, with reports of no significant impact or fewer hemodynamic changes, using 10% lidocaine^13^, 1% with a proportion of 9.5 mL lidocaine to 0.5 mL of sodium bicarbonate and 10 mL with 9 mL of 2% lidocaine+1 mL of lidocaine. However, there is higher SBP expression in the LG group than in the saline group, as reported by Soares^12^ and Carrillo, López, and González^11^.

Data also diverge from the findings of Oliveira^14^, determining whether tracheal tube cuffs filled with AL are related to a lower incidence of postoperative odynophagia and post-extubation hemodynamic changes in children undergoing tonsillectomy or adenotonsillectomy, not observing hemodynamic differences.

In our study, the CG had more individuals who could sustain phonation in 2 h than the ALG, which showed a significant increase in patients in 24 to 2 h. Individuals capable of sustaining adequate volume were more observed in the ALG than in the CG, which showed a significant increase in 24 h. There was a significant reduction in both groups regarding dysphonia, vocal fatigue, hoarseness, and whispering in 24 h compared to 2 h, but without significant differences between the groups. The ALG showed a higher percentage of foreign body sensation than the CG, with a significant reduction within 24 h. Both showed a slight, non-significant increase in cough complaints at 24 h compared to 2 h.

In contrast, Samperio-Guzmán et al.^15^ found that the presence of coughing during extubation was directly associated with the use of the saline solution in the transthoracic echocardiogram, increasing up to three times the probability of occurrence in the CG (odds ratio [OR] 3.3) and the lidocaine group presented an OR 0.3, being a protection factor with a 23% probability of coughing during extubation.

Studies corroborate our findings by demonstrating a reduction in other postoperative complaints with the use of AL in the inflation of the ETT cuff, such as throat and hoarseness^13,16^, in different circumstances of temperature, concentration, and procedure time. However, this reduction was also observed in the CG in our research.

Although the patient undergoing thyroidectomy may present signs, symptoms, and pre-thyroidectomy voice and swallowing alterations, they still exhibit laryngological alterations related to gastroesophageal reflux and the diagnosis of injury^17^.

The thyroidectomy can generate vocal and swallowing changes, resulting from several factors, such as the extension of the procedure, surgical technique, orotracheal intubation, dissection of cervical muscles and hematomas, and manipulation of laryngeal nerves^18^.

This study has limitations related to the lack of assessment of sensation and the associated preoperative discomfort. In addition, the sample includes older adults, which may influence the response regarding age-related difficulties. Also, we used a dilution of 2% lidocaine (2 mL)+8.4% sodium bicarbonate (4 mL), a lower dilution than other studies. Future research should include clinical and instrumental evaluation of swallowing and voice, symptoms, and discomfort before thyroidectomy and associate higher concentrations of AL to define an adequate standard for analgesia.

## CONCLUSION

In this study, the AL had no protective effect on post-thyroidectomy physiological reflexes to extubation, such as cough, time to awakening, speech recovery, and perioperative blood pressure levels. However, the hemodynamic response was significantly better after extubation in the intervention group. Nevertheless, the results suggest that this group of patients is an example of how potential preoperative signs and symptoms can affect the outcomes of endotracheal intubation.
